# Supplementary material for: Warning people that they are being microtargeted fails to eliminate persuasive advantage
Source: Commun Psychol. 2025 Jan 29;3:15. doi: 10.1038/s44271-025-00188-8 (PMC11774753; doi:10.1038/s44271-025-00188-8)
Supplement: Supplementary file 2 — Supplementary Information [file 44271_2025_188_MOESM2_ESM.pdf]

Supplementary Information: Warning people that they are being microtargeted fails to eliminate persuasive advantage

Fabio Carrella[1*], Almog Simchon[2], Matthew Edwards[3], Stephan Lewandowsky[1,4,5]

[1]School of Psychological Science, University of Bristol, Bristol, United Kingdom.
[2]Department of Psychology, Ben-Gurion University of the Negev, Beer Sheva, Israel.
[3]School of Computer Science, University of Bristol, Bristol, United Kingdom.
[4]Department of Psychology, University of Potsdam, Potsdam, Germany.

*Corresponding author(s). E-mail(s): fabio.carrella@bristol.ac.uk;

Supplementary Note 1

Below are reported the instructions displayed to participants across all three main studies, as well as the pilot study. These instructions appeared on screen just before the ads were shown and remained visible until the participant proceeded with the survey.

Instructions presented in the Pilot Study:

Please spend some time reading the information on this page. Then press the button below to start the experiment.

In the following task, you will be presented with a series of political advertisements that may have been published in the UK over the last 5 years. For each advertisement, you will be asked a few questions about the extent to which you find it appealing.

Please press the button below to start the experiment.

Instructions presented in Study 1:

Please spend some time reading the information on this page. Then press the button below to start the experiment.

In the following task, you will be presented with a series of political advertisements that may have been published in the UK over the last 5 years. For each advertisement, you will be asked a few questions about the extent to which you find it appealing.

Some of these advertisements may be tailored to specific characteristics, interests, or traits commonly associated with individuals. This process, known as microtargeting, involves tailoring advertisements to resonate with specific audience segments.

In this regard, we have included a popup before some advertisements to inform participants about the potential presence of targeting elements in the ad content.

Please press the button below to start the experiment.

Instructions presented in Studies 2a and 2b:

Please take some time to read the information on this page.

In the upcoming task, you will be presented a series of political advertisements that may have been published in the UK over the last 5 years. The ads only include text without images. Simultaneously, we will simulate a browser extension that, based on your responses to previous surveys, has the ability to identify political ads that target specific aspects of your personality. This targeting practice is known as 'psychological microtargeting.' Typically, microtargeting customizes content based on personal data, preferences, and online behavior. It becomes 'psychological' when it covertly infers psychological features from your data to tailor manipulative messages, often for political purposes or to spread misinformation.

Please note that the browser extension will analyze only the text of the ad, not its inherent message. It will display a pop-up warning when its algorithm detects that an ad you are viewing is designed to target your personality. Given that the algorithm is still in its early stages of development, the pop-up might not appear every time an ad is targeting your personality.

Finally, for each advertisement, you will be asked a few questions about the extent to which you find it appealing.

Click the button below to start the experiment.

Supplementary Note 2

Below is reported the content of the popup as shown in the pilot study and in Study 1:

Warning: *This advertisement may appear highly appealing or tailored specifically to you. If you come across such content, it's probably due to psychological microtargeting. Microtargeting customizes content based on your personal data, preferences, and online behavior. It becomes 'psychological' when it covertly infers psychological features from your data to tailor manipulative messages, often for political purposes or to spread misinformation.*

The popup text was revised in Study 2a and 2b as shown below. Namely, we moved the brief explanation about psychological microtargeting to the instructions, improving the popup readability. A rendering of how the popup appeared to participants is depicted in Supplementary Figure 1

Warning: *This advertisement appears to be written in a style tailored to appeal to you. If you come across such content, it's probably due to psychological microtargeting.*

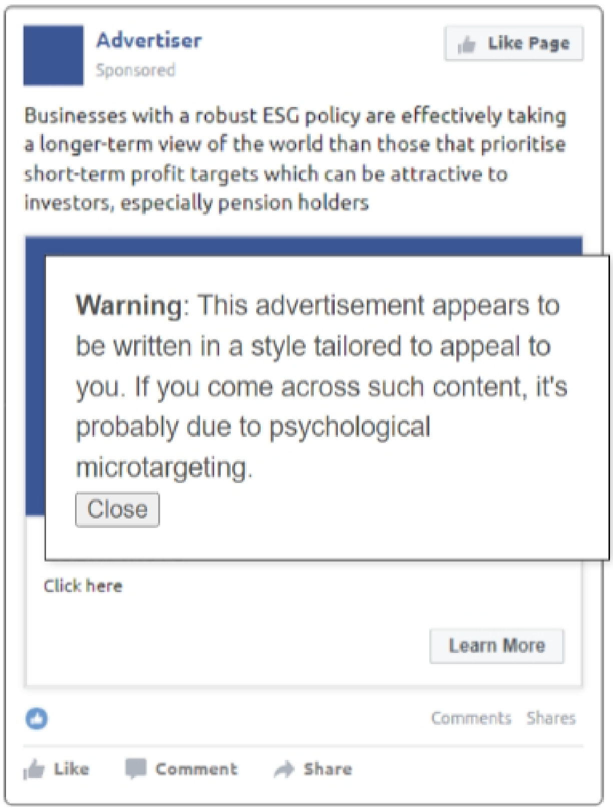

Supplementary Figure 1 Illustration of the on-screen appearance of the popup to Study 2a and Study 2b participants upon detecting a targeted ad. The popup remained visible until the participant clicked the close button.

Supplementary Note 3

In our pilot study, preregistered at https://aspredicted.org/p3am8.pdf, participants were divided into two groups and were equally exposed to five personality-targeted ads and five non-targeted ads. The intervention group saw targeted ads accompanied by a popup warning about the potential microtargeting nature of the ad. This popup also provided a brief explanation of psychological microtargeting and its associated dangers. It appeared superimposed on the ad and stayed on the screen until the participant clicked a close button. The control group was not presented with such warning.

Following [1], we fitted a linear mixed-effects model to predict self-reported persuasiveness with a two-way interaction between group (i.e., control vs. intervention) and a personality matching score. This score represents the absolute difference between the scaled openness scores of a $participant_i$ and an ad_j. The former were obtained from previous surveys we conducted on ads persuasiveness (reported in [1]), whereas the latter were calculated using the language model described in [2]. The final matching score was extracted as follows: $Matching_{i_j} = |z(Openness_i) - z(Openness_j)|$. By incorporating the matching score, we took into account each person's degree of openness. Also, we did not average persuasiveness rates within participants. Instead, we included a random intercept for the different participants to account for multiple observations, and random slopes and intercepts for ads to account for the fact that the same ad could appear under different conditions (e.g., with or without a popup).

Results show that the matching score was the only significant predictor ($\beta = -0.048$, $CI = [-0.077, -0.018]$, $p < 0.001$), indicating that perceived persuasiveness is higher when the participant's and the ad's openness are identical (i.e., $matching\ score = 0$), and decreases the more they differ. Neither the intervention, nor its interaction with the matching score, was significant, attesting to the ineffectiveness of the warning pop up. Further details of the regression are shown in Table Supplementary Table 1.

To test for the robustness of our non-significant effect of popups on persuasiveness, we performed a non-preregistered equivalence test using the *parameters* R package [3]. Since the effect of being in the intervention group is not statistically significant and the narrow confidence intervals (i.e., $1 - 2 * \alpha$, which equals to 90%) overlaps with the region of practical equivalence (ROPE), the equivalence is neither accepted nor rejected (90% CI = [-0.202, 0.002]). The ROPE for the equivalence tests was set to default bounds automatically defined based on our models' properties, calculated as $[-0.1 * SD$y, $0.1 * SD$y]. For this study, this corresponds to the range [-0.124, 0.124].

Supplementary Note 4

To gauge perceived persuasion, we revised the ad perceived persuasiveness scale [4] by incorporating six items: 1. "I find this ad to be persuasive"; 2. "This is an effective ad"; 3. "I would click on this link after seeing this ad"; 4. "Overall, I like this ad"; 5. "This ad has made me more interested in the topic"; 6. "I am interested in learning more about this topic after seeing this ad". Participants rated each ad's effectiveness using a 5-point Likert scale, ranging from "strongly disagree" to "strongly agree".

The scale demonstrated high internal consistency in a psychometric assessment. A multilevel reliability analysis [5] revealed a multilevel Cronbach's alpha of 0.93. Additionally, an exploratory factor analysis [1] suggested that a single factor best accounted for the variance structure of the items.

Supplementary Note 5

In the preregistration of Study 1, we declared our intention to conduct an analysis of the effect of the popup on the different items of the scale used, to ascertain whether the popup may have varying effects depending on the aspect measured. Consequently,

we performed a factor analysis to group the six items of the questionnaire into two distinct factors. The factor analysis, conducted with oblique rotation of the 'promax' type due to the correlation between the items, suggested the presence of two factors reported in Supplementary Table 2 and described as follows:

- Ad Interaction: This factor explained 41% of the variance and included the following items: "I would click on this link after seeing this ad", "This ad has made me more interested in the topic", and "I am interested in learning more about this topic after seeing this ad", with factor loadings ranging between 0.70 and 0.97.
- Ad Appeal: This factor explained 37% of the variance and included the following items: "I find this ad to be persuasive", "This is an effective ad", and "Overall, I like this ad", with factor loadings ranging between 0.60 and 0.96.

We conducted two regressions following the same design outlined in the main text. Specifically, we created two linear mixed models to predict the two factors, incorporating a two-way interaction between ad type and the matching score. We also included random intercepts for ads and participants to address multiple observations (i.e., the persuasiveness ratings) per subject. To accommodate the varying ranges of the matching score across ad types (i.e., non-targeted ads inherently are less likely to match a person's personality), we mean-centered the matching score, ensuring that the mean persuasiveness within each ad type is equal to 0.

The results of the regressions indicate that "ad appeal" decreases when ads are non-targeted compared to targeted ads without popups ($t(9, 990)$ = -5.676, $p < 0.001$, $\beta = -0.131$, 95% CI = [-0.176, -0.086]), when the matching score is at its mean. All other predictors, including the interaction between ad types and the mean-centered matching scores, were found to be non-significant. Conversely, when "ad interaction" was considered as the dependent variable, the model exhibited a significant decreasing effect of popups compared to targeted ads without popups ($t(9, 990)$ = -2.004, p = 0.045, $\beta = -0.042$, 95% CI = [-0.083, -0.001]). A larger decrease in "ad interaction" was observed for non-targeted ads compared to targeted ads without popups ($t(9, 990)$ = -3.206, $p < 0.001$, β = -0.075, 95% CI = [-0.121, -0.029]). No other predictors or interactions were deemed significant.

Subsequently, we conducted the same factor analysis and regressions on the data from Study 2a. The factor analysis results unveiled an almost identical factorial structure and loadings. However, the regression analysis yielded slightly different outcomes. When "ad appeal" was specified as the dependent variable, we found Study 2a to replicate Study 1 results, with non-targeted ads showing a negative effect on ad appeal ($t(6, 480)$ = -3.280, $p < 0.001$, β = -0.094, 95% CI = [-0.150, -0.038]). Conversely, when "ad interaction" was the dependent variable, we observed that the presence of a popup did not have a significant impact on the dependent variable anymore. Additionally, we found a significant effect of the mean-centered matching score ($t(6, 480)$ = -2.107, p = 0.035, β = -0.214, 95% CI = [-0.413, -0.015]), suggesting that targeted ads have a higher likelihood of increasing interaction with the ads themselves. Further details are reported in Supplementary Table 3 and Supplementary Table 4.

Supplementary Note 6

The models reported in our main paper differed from the ones described in our preregistration (Study 1: https://aspredicted.org/2je46.pdf, Study 2a was not preregistered as it shared the same identical study design and structure of Study 1). The preregistered analyses did not include random slopes to account for the effects of ad type between people and ads, considering that the same ad could appear under different ad types. Additionally, we decided post hoc to mean-center the matching score to better account for its different ranges across ad types, despite this operation not being preregistered. For full transparency, we report here the results of the original preregistered model, which included only random intercepts for participants and ads, as well as an untransformed matching score.

Results for the preregistered model of Study 1 show that the popup has a significant decreasing effect on persuasiveness ($t(59, 940)$ = -3.926, $p < 0.001$, β = -0.069, 95% CI=[-0.104, -0.035]). This contradicts the findings reported in our main paper, where popup ads did not show any significant effect in Study 1 and 2a. However, an exploratory equivalence test still shows that the effect of popups falls within the practical region of equivalence of [-0.126, 0.126], making it equivalent to the effects of targeted ads without popups (90% CI = [-0.098, -0.040]). This confirms the conclusion in the main manuscript that the popups have a negligible effect on persuasiveness. Finally, applying the same preregistered regression formula to the data from Study 2a does not reveal any significant effect of popup warnings ($t(38, 880)$ = -1.928, p = 0.054, β = -0.047, 95% CI=[-0.095, 0.001]), effectively confirming our main paper findings.

Supplementary References

[1] A. Simchon, M. Edwards, S. Lewandowsky, The persuasive effects of political microtargeting in the age of generative artificial intelligence. PNAS Nexus **3**(2), pgae035 (2024). https://doi.org/10.1093/pnasnexus/pgae035. URL https://academic.oup.com/pnasnexus/article/doi/10.1093/pnasnexus/pgae035/7591134

[2] A. Simchon, A. Sutton, M. Edwards, S. Lewandowsky, Online reading habits can reveal personality traits: towards detecting psychological microtargeting. PNAS Nexus **2**(6), pgad191 (2023). https://doi.org/10.1093/pnasnexus/pgad191. URL https://academic.oup.com/pnasnexus/article/doi/10.1093/pnasnexus/pgad191/7191531

[3] D. Lüdecke, M.S. Ben-Shachar, I. Patil, D. Makowski, Extracting, computing and exploring the parameters of statistical models using R. Journal of Open Source Software **5**(53), 2445 (2020). https://doi.org/10.21105/joss.02445

[4] J.B. Hirsh, S.K. Kang, G.V. Bodenhausen, Personalized persuasion: tailoring persuasive appeals to recipients' personality traits. Psychological Science **23**(6), 578–581 (2012). https://doi.org/10.1177/0956797611436349. URL http://journals.sagepub.com/doi/10.1177/0956797611436349

[5] J.B. Nezlek, A practical guide to understanding reliability in studies of within-person variability. Journal of Research in Personality **69**, 149–155 (2017). https://doi.org/10.1016/j.jrp.2016.06.020. URL https://linkinghub.elsevier.com/retrieve/pii/S009265661630068X

Supplementary Table 1 Results of the generalised linear mixed-effects model used in the pilot study, with the reported persuasiveness as dependent variable. Estimates are presented together with their 95% confidence intervals. In this model, the observations (i.e., the persuasiveness rates) are considered individually rather than being averaged within subjects. To address the fact that each participant rated multiple ads on various scale items, and that the same ads appeared under different conditions, both participants and ads were designated as random effects in the analysis. Additionally, the ad type effect within each ad was accounted for by including random slopes for ad type. Further variables considered in the model include group assignment (control or intervention) and the matching score, which represents the scaled difference between the participant's openness score and that of the ad. Both group type and the matching score are included in the formula as a two-way interaction.

	Dependent variable:
	Persuasiveness
intervention	−0.100
	(-0.222,0.022)
matching_score	−0.048**
	(-0.077,-0.018)
intervention:matching_score	0.022
	(-0.015,0.060)
Constant	2.678***
	(2.555,2.801)
Observations	33,900
Log Likelihood	-51,034.440
Akaike Inf. Crit.	102,092.900
Bayesian Inf. Crit.	102,194.000
Note:	*p<0.05; **p<0.01; ***p<0.001

Supplementary Table 2 Factor loadings derived from the factorial analysis of the persuasiveness items utilized in Study 1.

	Factor 1	Factor 2
I find this ad to be persuasive	-0.004	0.938
This is an effective ad	-0.014	0.955
I would click on this link after seeing this ad	0.703	0.210
Overall, I like this ad	0.310	0.599
This ad has made me more interested in the topic	0.949	0.028
I am interested in learning more about this topic after seeing this ad	0.971	0.004

Supplementary Table 3 Results of the generalised linear mixed-effects model described used in Study 1 (left) and Study 2a (right), with the "ad appeal" factor as dependent variable. Estimates are reported together with their 95% confidence intervals. In both models, the observations (i.e., the factorial scores) are reported individually rather than being averaged within subjects. To address the fact that each participant rated multiple ads on various scale items, both participants and ads were designated as random effects in the analysis. Additional variables considered in the model include ad type ('targeted-no popup', used as reference level, 'non-targeted', and 'targeted popup') and the matching score, which has been mean-centered within each ad type and represents the scaled difference between the participant's openness score and that of the ad. Both ad type and the matching score are included in the formula as a two-way interaction.

	Dependent variable:	
	Ad Appeal	
	(Study 1)	(Study 2)
popup	0.019	−0.016
	(-0.021,0.059)	(-0.067,0.036)
non_targeted	−0.131***	−0.094**
	(-0.176,-0.086)	(-0.150,-0.038)
matching_score_centered	0.004	0.091
	(-0.121,0.130)	(-0.107,0.289)
popup:matching_score_centered	0.018	−0.003
	(-0.137,0.173)	(-0.235,0.229)
non_targeted:matching_score_centered	−0.066	−0.171
	(-0.202,0.070)	(-0.387,0.044)
Constant	0.040	0.038
	(-0.072,0.152)	(-0.059,0.134)
Observations	9,990	6,480
Log Likelihood	-13,038.990	-8,614.250
Akaike Inf. Crit.	26,095.990	17,246.500
Bayesian Inf. Crit.	26,160.870	17,307.490
Note:	*p<0.05; **p<0.01; ***p<0.001	

Supplementary Table 4 Results of the generalised linear mixed-effects model described used in Study 1 (left) and Study 2a (right), with the "ad interaction" factor as dependent variable. Estimates are reported together with their 95% confidence intervals. In both models, the observations (i.e., the factorial scores) are reported individually rather than being averaged within subjects. To address the fact that each participant rated multiple ads on various scale items, both participants and ads were designated as random effects in the analysis. Additional variables considered in the model include ad type ('targeted-no popup', used as reference level, 'non-targeted', and 'targeted popup') and the matching score, which has been mean-centered within each ad type and represents the scaled difference between the participant's openness score and that of the ad. Both ad type and the matching score are included in the formula as a two-way interaction.

	Dependent variable:	
	Ad Interaction	
	(Study 1)	(Study 2)
popup	−0.042*	−0.020
	(-0.083,-0.001)	(-0.073,0.032)
non_targeted	−0.075**	−0.124***
	(-0.121,-0.029)	(-0.182,-0.067)
matching_score_centered	−0.086	−0.214*
	(-0.213,0.042)	(-0.413,-0.015)
popup:matching_score_centered	0.122	0.067
	(-0.036,0.280)	(-0.170,0.303)
non_targeted:matching_score_centered	−0.069	0.061
	(-0.207,0.070)	(-0.157,0.279)
Constant	0.039	0.048
	(-0.060,0.139)	(-0.061,0.157)
Observations	9,990	6,480
Log Likelihood	-13,214.160	-8,698.205
Akaike Inf. Crit.	26,446.320	17,414.410
Bayesian Inf. Crit.	26,511.200	17,475.400
Note:	*p<0.05; **p<0.01; ***p<0.001	

Supplementary Table 5 Results of the preregistered generalised linear mixed-effects model used in the pilot study, with the reported persuasiveness as dependent variable. Estimates are presented together with their 95% confidence intervals. In this model, the observations (i.e., the persuasiveness rates) are considered individually rather than being averaged within subjects. To address the fact that each participant rated multiple ads on various scale items, we specified random intercepts for both participants and ads. Additional variables considered in the model include group assignment (control or intervention) and the matching score, which represents the scaled difference between the participant's openness score and that of the ad. Both group type and the matching score are included in the formula as a two-way interaction.

	Dependent variable:
	Persuasiveness
intervention	-0.090
	(-0.201,0.022)
matching_score	-0.049^{***}
	(-0.069,-0.028)
intervention:matching_score	0.016
	(-0.012,0.044)
Constant	2.687^{***}
	(2.567,2.807)
Observations	33,900
Log Likelihood	-51,064.100
Akaike Inf. Crit.	102,142.200
Bayesian Inf. Crit.	102,201.200
Note:	*p<0.05; **p<0.01; ***p<0.001

Supplementary Table 6 Results of the preregistered generalised linear mixed-effects model used in Study 1 (left) and Study 2a (right), with reported persuasiveness as dependent variable. Estimates are reported together with their 95% confidence intervals. In both models, the observations (i.e., the persuasiveness ratings) are reported individually rather than being averaged within subjects. To address the fact that each participant rated multiple ads on various scale items, we specified random intercepts for both participants and ads. Additional variables considered in the model include ad type (with 'targeted-no popup' used as reference level) and the matching score, which represents the scaled difference between the participant's openness score and that of the ad. Both ad type and the matching score are included in the formula as a two-way interaction.

	Dependent variable:	
	Persuasiveness	
	(Study 1)	(Study 2)
popup	-0.069^{***}	-0.047
	(-0.104,-0.035)	(-0.095,0.001)
non_targeted	0.110^{**}	0.130^{*}
	(0.027,0.193)	(0.012,0.247)
matching_score	-0.019	-0.132^{*}
	(-0.086,0.047)	(-0.240,-0.025)
popup:matching_score	0.128^{**}	0.046
	(0.049,0.208)	(-0.074,0.166)
non_targeted:matching_score	-0.130^{***}	-0.052
	(-0.200,-0.059)	(-0.167,0.063)
Constant	2.695^{***}	2.792^{***}
	(2.576,2.814)	(2.664,2.921)
Observations	59,940	38,880
Log Likelihood	-89,176.570	-59,078.690
Akaike Inf. Crit.	178,371.100	118,175.400
Bayesian Inf. Crit.	178,452.200	118,252.500
Note:	*p<0.05; **p<0.01; ***p<0.001	
