# Supplementary material for: Warning people that they are being microtargeted fails to eliminate persuasive advantage
Source: Commun Psychol. 2025 Jan 29;3:15. doi: 10.1038/s44271-025-00188-8 (PMC11774753; doi:10.1038/s44271-025-00188-8)
Supplement: Supplementary file 3 — Reporting Summary [file 44271_2025_188_MOESM3_ESM.pdf]

nature portfolio

Corresponding author(s): Fabio Carrella

Last updated by author(s): Dec 18, 2024

Reporting Summary

Nature Portfolio wishes to improve the reproducibility of the work that we publish. This form provides structure for consistency and transparency in reporting. For further information on Nature Portfolio policies, see our Editorial Policies and the Editorial Policy Checklist.

Statistics

For all statistical analyses, confirm that the following items are present in the figure legend, table legend, main text, or Methods section.

n/a	Confirmed	
☐	☒	The exact sample size (*n*) for each experimental group/condition, given as a discrete number and unit of measurement
☐	☒	A statement on whether measurements were taken from distinct samples or whether the same sample was measured repeatedly
☐	☒	The statistical test(s) used AND whether they are one- or two-sided<br>*Only common tests should be described solely by name; describe more complex techniques in the Methods section.*
☐	☒	A description of all covariates tested
☐	☒	A description of any assumptions or corrections, such as tests of normality and adjustment for multiple comparisons
☐	☒	A full description of the statistical parameters including central tendency (e.g. means) or other basic estimates (e.g. regression coefficient) AND variation (e.g. standard deviation) or associated estimates of uncertainty (e.g. confidence intervals)
☐	☒	For null hypothesis testing, the test statistic (e.g. *F*, *t*, *r*) with confidence intervals, effect sizes, degrees of freedom and *P* value noted<br>*Give P values as exact values whenever suitable.*
☒	☐	For Bayesian analysis, information on the choice of priors and Markov chain Monte Carlo settings
☐	☒	For hierarchical and complex designs, identification of the appropriate level for tests and full reporting of outcomes
☐	☒	Estimates of effect sizes (e.g. Cohen's *d*, Pearson's *r*), indicating how they were calculated

Our web collection on statistics for biologists contains articles on many of the points above.

Software and code

Policy information about availability of computer code

Data collection	Data was collected through the online survey platform Prolific.com. Participants on Prolific were administered a survey created using the survey platform Qualtrics.com.
Data analysis	Data were analyzed using custom R (v.4.3.0) scripts. Specifically, we used the following third-party R packages: tidyverse (2.0.0), rstatix (0.7.2), lme4 (1.1-33), sjPlot (2.8.15), interactions (1.1.5), psych (2.3.6), simr (1.0.7), marginaleffects (0.20.1), and parameters (0.21.3). The code for the data analysis is available on OSF (https://osf.io/rxhtc/)

For manuscripts utilizing custom algorithms or software that are central to the research but not yet described in published literature, software must be made available to editors and reviewers. We strongly encourage code deposition in a community repository (e.g. GitHub). See the Nature Portfolio guidelines for submitting code & software for further information.

Data

Policy information about availability of data

All manuscripts must include a data availability statement. This statement should provide the following information, where applicable:

- Accession codes, unique identifiers, or web links for publicly available datasets
- A description of any restrictions on data availability
- For clinical datasets or third party data, please ensure that the statement adheres to our policy

The data collected for the studies reported in the main manucript as well as the supplement are available on OSF (https://osf.io/rxhtc/). For privacy reasons, we

have substituted the Prolific IDs of all participants across the three studies with random alphanumeric IDs. This measure ensures the anonymity and confidentiality of the participants' identities. Importantly, this substitution does not affect the reproducibility or validity of the analysis conducted in the studies.

Human research participants

Policy information about studies involving human research participants and Sex and Gender in Research.

Reporting on sex and gender	This information is reported in the Methods section.
Population characteristics	This information has not been collected.
Recruitment	Our samples for Study 1 and 2a were drawn from pools of subjects who had participated in prior surveys related to ad persuasiveness, as described in Simchon et al. (2024). In these previous surveys, we measured participants' levels of openness to new experiences. For Study 2b, we instead used a new sample of participants who had never been exposed to any of our previous experiments.<br><br>For all three studies, we selected individuals from each pool who fell in either the top or bottom 30% of openness scores. This selection allowed us to unambiguously target ads (i.e., low- or high-openness) to match their personality. To ensure that only these selected participants were included in our studies, we created an approved list of their IDs when setting up the study on Prolific.
Ethics oversight	The School of Psychological Science Research Committee at the University of Bristol provided ethics approval for the experiments (ethics approvals #17566, and #17923)

Note that full information on the approval of the study protocol must also be provided in the manuscript.

Field-specific reporting

Please select the one below that is the best fit for your research. If you are not sure, read the appropriate sections before making your selection.

☐ Life sciences ☒ Behavioural & social sciences ☐ Ecological, evolutionary & environmental sciences

For a reference copy of the document with all sections, see nature.com/documents/nr-reporting-summary-flat.pdf

Behavioural & social sciences study design

All studies must disclose on these points even when the disclosure is negative.

Study description	This research consists of three quantitative experiments investigating the effects of microtargeting in political advertising and the potential impact of warning signals. All three studies employ within-subjects designs. The research collects and analyzes quantitative data, measuring the persuasiveness of targeted versus non-targeted political ads under different conditions (with and without warning popups). The studies use statistical methods to assess the effectiveness of personality-tailored political ads and evaluate whether warning signals significantly impact their persuasiveness.
Research sample	For Study 1 and 2a, we recruited participants from a pool of individuals who had previously taken part in surveys on ad persuasiveness, as detailed in Simchon et al. (2024). In these earlier surveys, participants' openness levels were measured using the BFI-2 (Soto & John, 2017). For Study 2b, we instead used a new sample of participants who had never been exposed to any of our previous experiments. In this case, we also measured participants' openness levels using items from the BFI-2.
Sampling strategy	We sampled individuals scoring in either the top or bottom 30% of our entire research sample, allowing us to match ads (high- or low-openness) to their personality traits. We created an approved list of participant IDs on Prolific to ensure only these selected individuals participated in our studies.
Data collection	Data was collected through the online survey platform Prolific.com. Participants on Prolific were administered a survey created using the survey platform Qualtrics.com.
Timing	Data collection for Study 1, Study 2a and Study 2b started on December 13, 2023, February 8, 2024, and October 21, 2024, respectively. In all three cases, data collection ended 24 hours after the starting date.
Data exclusions	We excluded participants based on two criteria: those who completed the survey in a time that was more than three standard deviations below the mean completion time, and those who provided the same exact answer (on a scale from 1 to 5) throughout the entire survey. These exclusion criteria were designed to ensure data quality by removing responses that were likely to be inattentive or invalid.
Non-participation	In the survey instructions, we explicitly informed participants that they could withdraw from the study at any time by simply closing their browser. Due to technical limitations, we were only able to record data for participants who completed the survey. As a result, we do not have information on the number of participants who chose to drop out before completion.

Randomization	Based on their openness levels, participants were directed to either a low- or high-openness version of the survey. Regardless of the version, all participants completed the same task: rating the persuasiveness of presented ads. In both studies, warning popups appeared before 5 out of the 10 targeting ads. The presentation of these popups was randomized using a custom JavaScript script implemented in Qualtrics.

Reporting for specific materials, systems and methods

We require information from authors about some types of materials, experimental systems and methods used in many studies. Here, indicate whether each material, system or method listed is relevant to your study. If you are not sure if a list item applies to your research, read the appropriate section before selecting a response.

Materials & experimental systems

n/a	Involved in the study
☒	☐ Antibodies
☒	☐ Eukaryotic cell lines
☒	☐ Palaeontology and archaeology
☒	☐ Animals and other organisms
☒	☐ Clinical data
☒	☐ Dual use research of concern

Methods

n/a	Involved in the study
☒	☐ ChIP-seq
☒	☐ Flow cytometry
☒	☐ MRI-based neuroimaging
